# “Pre-schoolers in the playground” an outdoor physical activity intervention for children aged 18 months to 4 years old: study protocol for a pilot cluster randomised controlled trial

**DOI:** 10.1186/1745-6215-14-326

**Published:** 2013-10-09

**Authors:** Sally E Barber, Cath Jackson, Shaheen Akhtar, Daniel D Bingham, Hannah Ainsworth, Catherine Hewitt, Gerry Richardson, Carolyn D Summerbell, Kate E Pickett, Helen J Moore, Ash C Routen, Claire L O’Malley, Shirley Brierley, John Wright

**Affiliations:** 1 Born in Bradford Cohort Study, Bradford Institute for Health Research, Bradford, UK; 2 Department of Health Sciences, University of York, York, UK; 3 School of Sport Exercise and Health Sciences, Loughborough University, Loughborough, UK; 4 Centre for Health Economics, University of York, York, UK; 5 School of Medicine and Health, University of Durham, Durham, UK; 6 School of Education, University of Durham, Durham, UK; 7 Public Health, City of Bradford Metropolitan District Council, Bradford, UK

**Keywords:** Pre-school, Physical activity, Intervention, Feasibility, Early years

## Abstract

**Background:**

The pre-school years are considered critical for establishing healthy lifestyle behaviours such as physical activity. Levels of physical activity track through childhood into adulthood, thus establishing habitual physical activity early in life is vital. Time spent outdoors is associated with greater physical activity and playground interventions have been shown to increase physical activity in school aged children. There are few pre-school, playground-based interventions, and evaluations of these have found mixed results. A recent report published by the UK Chief Medical Officer (CMO) highlighted that new interventions to promote movement in the early years (0–5 years old) are needed. The aim of this study is to undertake a pilot cluster randomised controlled trial (RCT) of an outdoor playground-based physical activity intervention for parents and their children aged 18 months to 4 years old (“Pre-schoolers in the Playground”; PiP) and to assess the feasibility of conducting a full scale cluster RCT. The PiP intervention is grounded in behavioural theory (Social Cognitive Theory), and is in accordance with the CMO guidance for physical activity in the early years. It is informed by existing literature and data collected from focus groups with parents.

**Methods/Design:**

One hundred and fifty pre-school children affiliated to 10 primary schools will be recruited. Schools will be randomised to either the PiP intervention arm or the control arm (usual practice). Children in the intervention arm will be invited to attend three 30 minute outdoor play sessions per week for 30 weeks (3 school terms) at the school. Feasibility will be assessed by examining recruitment rates, attendance, attrition, acceptability of the trial and of the PiP intervention to parents, fidelity of intervention implementation, capability and capacity for schools to deliver the intervention. Health outcomes and the feasibility of outcome measurement tools will be assessed. These include physical activity via triaxial, accelerometry (Actigraph GT3X+), anthropometry (height, body mass, BMI, waist and upper arm circumference), health related quality of life for child (PedsQL) and parent (EQ5D), parent wellbeing (ComQol-A5), injuries and health service use. A health economic evaluation will also be undertaken.

**Discussion:**

It is anticipated that results of this pilot trial will be published in spring 2015.

**Trial registration:**

Current controlled trials: ISRCTN54165860

## Background

### Physical activity and the pre-school years

The pre-school years are considered a critical period for establishing healthy lifestyle behaviours such as physical activity [1]. The benefits of engaging in regular physical activity in the pre-school years are numerous, with one of the most significant being the promotion of healthy weight and prevention of obesity during childhood [2-5]. In pre-school children the prevalence of overweight and obesity has doubled in recent decades [6] and in the mid-2000s over a third of pre-school children in the UK and US were overweight and obese [7]. Despite the widespread belief that the prevalence of childhood obesity is still escalating, contemporary high-quality studies suggest a slowing in the rate of rise in some developed countries including the UK [8,9]. Whilst this appears promising, levels still remain high and are heterogeneous within countries [8]. For example, in England childhood obesity is higher in urban areas, in children from deprived backgrounds and in certain ethnic minority groups, including Black and Asian populations [10]. Also, South Asian school-aged children are reported to have substantially lower levels of physical activity than White Europeans [11]. These particularly low levels may contribute to the increased risk of obesity, coronary heart disease and diabetes seen in South Asians living in the UK [12].

Observational and experimental studies have shown that regular physical activity has other important health and social implications for pre-school children. Physical activity is valuable for developing motor skills, enhancing bone and muscle development and for social competence [13,14]. Furthermore, regular physical activity in this age group may also have beneficial effects upon cardiovascular disease risk factors including more favourable blood pressure and blood lipids [14-16]. Finally, levels of physical activity track through early childhood [17] and into adulthood, [18,19] thus, establishing habitual physical activity early in life may be key to remaining active throughout the lifespan.

Current levels of habitual physical activity in pre-school children in the UK and internationally are unclear. Methodological differences in the objective measurement of physical activity have resulted in a wide variation of levels reported. Daily physical activity levels have been reported to be as low as 90 minutes in the UK [20], with higher levels of 127 minutes per day reported in Belgium, Australia and the USA, [21-23] and 402 minutes in Portugal and the USA [22,24]. This large variation in reported physical activity behaviour is likely due to the application of different intensity cutpoints to accelerometry data (that is, cutpoint non-equivalence [25]) between different research groups, rather than actual behavioural differences between countries. Despite the lack of clarity regarding the extent of physical activity that pre-schoolers engage in, it has been clearly identified that health-enhancing physical activity declines markedly during childhood [26], with this decline potentially beginning in the early years [27]. Therefore the promotion of physical activity in the pre-school years is critical for the prevention of this age-related decline.

### UK physical activity policy for pre-school children

The importance of engaging pre-school children in daily physical activity was brought to the forefront in July 2011 with the publication of the UK’s first physical activity guidelines for children under 5-years-old in the Chief Medical Officer’s (CMO) report *start active, stay active*[28]. The report recommends 180 minutes of physical activity (light, moderate and vigorous intensity) each day, and states that the volume of physical activity is more important than the intensity. Physical activity should be spread throughout the day and include active play (activities that involve movements of all the major muscle groups), and the development of locomotor, stability and object-control skills.

These guidelines are a significant step in recognising the importance of physical activity promotion for pre-school children; however, guidelines themselves do not change behaviour. The determinants of physical activity in young children are unclear; one systematic review that studied correlates of pre-schoolers’ physical activity found that boys were more active than girls and that parents’ levels of physical activity and a child’s time spent outdoors had a positive association with physical activity [29]. Additionally, preliminary findings from an ongoing systematic review suggest that providing easy-access environments that facilitate physical activity are important [30]. The CMO report states that a ‘concerted and committed action to create environments and conditions that make it easier for people to be more active is needed [28, page 8]. The guidelines highlight the need for activities to promote movement in the early years and recognise that local communities can have a strong influence on behaviour. The report suggests investment in community level programmes in settings such as school playgrounds. We therefore aim to develop and evaluate such an intervention in an area of ethnic diversity and social deprivation in West Yorkshire, UK.

### Components of successful interventions

There are several features of successful interventions identified in the literature, which have informed the intervention reported here.

#### A) Theoretical underpinning

The utility of basing health promotion interventions upon sound theoretical frameworks is well-expounded [31]. A systematic review [32] conducted by members of our team (CDS, HJM) reported that the predominant behavioural change theory used in successful childhood obesity prevention interventions is Bandura’s social cognitive theory (SCT) [33,34]. This theory describes behavioural change as an interaction between personal*,* environmental and behavioural factors. Personal and environmental factors provide the framework for understanding behaviour. Some of the personal concepts include skills, self-efficacy, self-control and outcome expectancies, whereas environmental concepts include availability (for example, provision of space for physical activity) and opportunity for social support (for example, a group setting). The review also reported that providing information on behaviour-health links appeared to be an important component in childhood obesity prevention/physical activity interventions.

#### B) An outdoor setting

Enhancing environmental and cultural practices that support children to be more active throughout the day are thought to be promising strategies to prevent childhood obesity, particularly if the children perceive them as being fun [35]. Time spent outdoors correlates with physical activity levels in pre-school children [29] and outdoor play is associated with a lower risk of being overweight [36]. Indeed, in school-aged children, outdoor playground interventions have increased physical activity [37,38]. Furthermore, factors associated with increased moderate to vigorous physical activity (MVPA) in playground interventions are greater provision of equipment [37,38] and greater play space per child [39]. Interventions in pre-school playgrounds supervised by pre-school teachers, however, have had mixed success. Adding portable play equipment in a US pre-school playground increased physical activity levels in 3- to 5-year-olds [40]. In contrast, in Belgium, no change in physical activity levels of 4- and 5-year-olds was reported after providing playground markings, or play equipment or both in the pre-school playground [41]. The authors concluded that creating an activity-friendly environment may not be sufficient to promote physical activity in pre-schoolers and regular infusions of different equipment with more guidance and encouragement from adults to play in an active way is required. Currently there is no evidence regarding pre-school playground interventions from the UK.

#### C) Parental involvement

Parental engagement should be a key part of any intervention in the pre-school years [42] and the suggestion that adult encouragement is required to increase active play is supported by findings from a systematic review of pre-school obesity prevention interventions [43]. Twelve interventions reported in another obesity prevention systematic review were conducted in a variety of settings (pre-school/childcare, home, group, primary care and mixed settings) and included a physical activity component. Home-based interventions appeared to be the most successful at increasing physical activity despite the small sample sizes of studies and poor adherence to the interventions. This is perhaps due to parental involvement in the interventions, which has been suggested to be vital for facilitating behavioural change during the early years [32]. Mothers in particular are considered to play a major role in establishing a child’s health lifestyle behaviour [44], and this in part may be indirectly through modelling of behaviours [45].

### Aim

The aim of this paper is to describe the protocol, Pre-schoolers in the Playground (PiP), for a pilot cluster randomised controlled trial (RCT) of an outdoor playground-based physical activity intervention for parents and their children aged 18 months to 4 years, which will assess the feasibility of conducting a full-scale cluster RCT.

## Design and methods

### Study design

**Figure 1:**
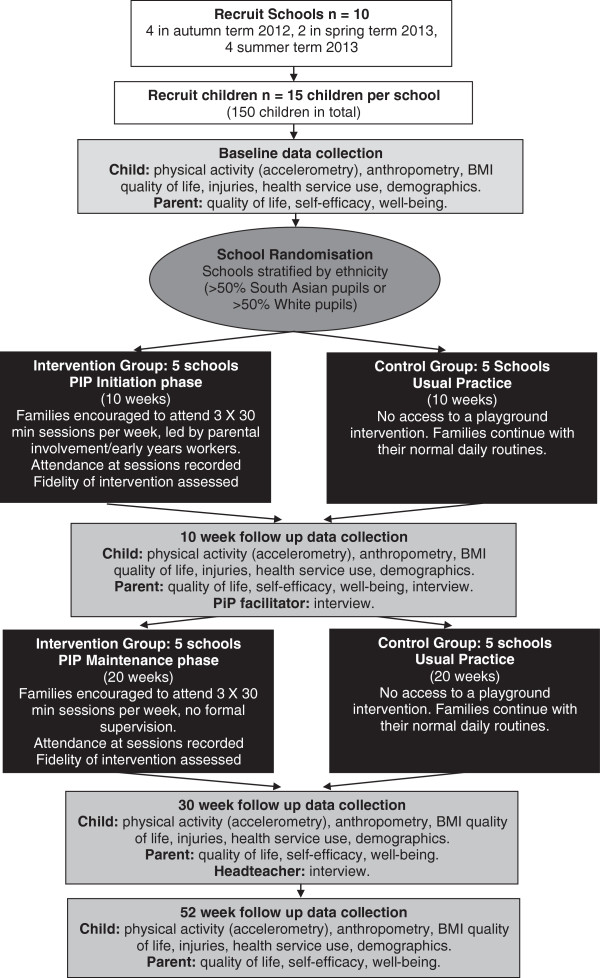
**“Pre-schoolers in the playground” (PIP) pilot trial flow-diagram.** BMI, body mass index.

The design of this pilot study is based on guidance from the UK Medical Research Council for developing and evaluating complex interventions [46]. Figure 1 shows the trial design; the trial is a two-armed pilot cluster randomised controlled trial with economic and qualitative evaluations. The two arms are PiP intervention and usual practice (control). Recruitment, randomisation and the intervention or control period will take place in three waves. Wave 1 commenced in the autumn 2012 school term, Wave 2 commenced in the spring 2013 school term and Wave 3 will commence in the summer 2013 school term. This staggered approach will allow the examination of seasonal variations in the feasibility of the study and the intervention. The intervention comprises a 10-week initiation phase (one school term) followed by a 20-week maintenance phase (two school terms). Follow up will be at 10 weeks, (mid-intervention), 30 weeks (at the end of the intervention) and 52 weeks. This study has been ethically approved by the NRES Committee Yorkshire and The Humber - Bradford (reference: 12/YH/0334) and complies with the Declaration of Helsinki.

### Setting

The setting for the trial is Bradford, West Yorkshire, UK. Bradford is the sixth largest metropolitan borough in England with a population of approximately 500,000 and includes some of the most deprived areas in the UK [47]. The national child measurement programme for 2010 to 2011 reports that 22% of children in reception and 35% of children in Year 6 in Bradford are overweight or obese [10]. Additionally, the city has very low levels of participation in school sport [48]. Half of all of babies born in Bradford are of South Asian origin (mainly of Pakistani and Bangladeshi heritage) [49] and 60% of these are born into the poorest 20% of the population of England and Wales based on the index of multiple deprivation (IMD) [47].

### School recruitment

Ten primary schools will be recruited from the 122 primary schools in Bradford, between September 2012 and April 2013. Two primary schools will have associated Children's Centres located on the school site. This is so that the feasibility of conducting the study either at a primary school only, or a joint primary school and children’s centre can be assessed.

Primary schools have been chosen as the site for intervention delivery because of their strong links with other early years services and in order to engage with children from hard-to-reach families who have an older sibling attending the school and do not attend any other early years services. In England early years services include nursery classes held on a primary school site, private day nurseries, pre-schools, independent schools, child minders and Children’s Centres. Children’s Centres are often government funded (Sure Start Children’s Centres). They provide support for young children and families and are particularly focused on the most disadvantaged families, in order to reduce inequalities in child development and school readiness. Children’s Centres work closely with other early years services (nurseries and schools); they can be located on the same site as other services (that is, on the same site as a primary school), or at a separate site.

The study was initially publicised to primary schools though advertising and presentations at education conferences and events hosted for Bradford primary school staff. Recruitment will be through telephone contact and visits to schools. Each study site will complete a study site agreement, which will be signed by the Head teacher and Chair of Governors. Schools that are located in the two poorest quintiles of IMD for Bradford, and that are located across the city of Bradford (Bradford South, West, East and Shipley) will be selected. We estimate, using the *English Indices of Deprivation* 2010 for Bradford District [47], that >80% of schools in Bradford will fall into the two poorest quintiles of IMD. To express thanks for participating in the pilot trial, all schools will receive a donation of £200 towards play equipment. Because the children are pre-school age and not attending the primary school, those affiliated to the schools in the control arm will not have access to the play equipment during the trial period.

### Participant recruitment

Families from participating schools and families who use children’s services that are affiliated with the school (for example, Children’s Centres or nurseries feeding those schools) will be approached to take part in the trial. Recruitment will be via letters home with school-going children and through face-to-face conversations with research assistants in school playgrounds and Children’s Centres or nurseries. To account for the linguistic diversity among the study population, research staff recruiting families and conducting measurements and questionnaires will be bilingual and will undertake these tasks in either English or Urdu [49]. Urdu is the language spoken and understood by Pakistanis. It is also spoken and understood in many parts of Bangladesh. For Bangladeshi participants who do not fully understand Urdu, the consent forms will also be audio-recorded in Syleti and participants will be asked to sign a paper copy of the consent form (translations and transliterations will be prepared in collaboration with staff involved in the cohort study, Born in Bradford, according to standard Born-in-Bradford procedures) [49]. Written informed consent will be gained from all participants at the beginning of the trial. All families will receive a £10 voucher for a children’s shop as a thank you for attending each study measurement session (baseline, 10-, 30- and 52-week follow up) and for participating in a qualitative interview.

### Inclusion criteria

Children will be included if they are aged between 18 months and 4 years and are available to complete three school terms of the intervention before going into Reception class (first year of school).

### Exclusion criteria

Children will be excluded if their parent is unable to provide informed consent.

### Randomisation and blinding

Randomisation will be carried out by York Trials Unit Randomisation Service using a secure computer system. Once baseline data have been collected, participating schools will be randomly allocated to one of the two arms, PiP intervention or usual care (control), on an equal basis (that is, five schools in each arm). Since ethnicity may be an important predictor of physical activity [11] and thus, to avoid chance imbalance in ethnicity, schools will be stratified and blocked by ethnicity (> 50% South Asian pupils or > 50% White pupils).

Blinding of the trial coordinator, schools, participants, and parent-involvement workers/early-years staff will not be possible. The community research assistants who will be assessing outcome measures will be blinded to each school’s allocation as will the statistician and health economists performing the data analyses.

### Sample size

The study will aim to recruit 150 children from 10 schools (15 children at each school site), thus, five schools with a total of 75 children in the PiP intervention arm and five schools with a total of 75 children in the control arm. At least four clusters per arm are recommended for a cluster RCT [50] and this sample size exceeds recommendations for pilot trials [51]. Therefore the sample will be sufficiently large to provide clear estimates of recruitment and follow up.

### Trial arms

#### Usual practice arm (control)

Families allocated to the usual practice arm will not have access to a playground intervention and will continue with their daily routines as usual.

### PiP intervention arm

#### Development of the intervention

The UK CMO's guidance for physical activity in the under 5-year-olds [28] and existing literature was reviewed [32,41-43]. The predominant behavioural change theory used in successful childhood obesity prevention interventions [24] was Bandura’s SCT [33,34], therefore, the SCT was used to direct the intervention development. Additionally, six focus groups with a total of 17 White and Pakistani mothers and caregivers (English and Urdu speaking) explored typical physical activity in toddlers and the barriers to and facilitators for physical activity [52]. Mothers reported that their children were innately active and enjoyed playing outside. Lack of time was a major barrier to physical activity. Mothers minimised the number of journeys they took and were put off organised activity sessions, finding it inconvenient to leave the house with young children. Other barriers to outdoor physical activity included feeling that their neighbourhood was an unsafe place to play outdoors and needing help from another adult to take the children to the park. Additionally the season, weather, and having to take public transport to parks were also identified as barriers. Some key facilitators for physical activity were having someone to help during activities and having activities available locally. There was little variation in the reported barriers and facilitators between ethnicities. Using these sources of information, a manual and materials to deliver PiP sessions were developed. These were reviewed by five early-years workers and minor revisions were made.

### Delivery of the intervention

Primary school/Children's Centre playgrounds will be made available to parents and pre-school children at times when parents are likely to be attending school (for example, dropping off or picking up older children from school, or young children from nursery). These times have been chosen in order to capitalise on times of the day that parents are already out of the house and will minimise the number of journeys to activities that parents have to make (identified as a barrier in focus groups). Six PiP sessions per week for 30 weeks (three school terms) will be available to families. This number of sessions was chosen to meet the need for flexibility, which was highlighted at the focus groups. Families will be encouraged to attend at least three of these sessions each week. Each PiP session will last for 30 minutes and include play activities likely to be of light activity and MVPA. There is some evidence to suggest that as little as an extra ten minutes per day of MVPA significantly reduces fat mass [2]. Furthermore, 56 minutes of MVPA per day in boys and 42 minutes in girls aged 5 to 8 years has been reported to improve metabolic status assessed from a composite score of various cardiovascular disease risk factors [53]. Given the sporadic nature of physical activity in these very young children, and given that between 25 and 70% of total physical activity in pre-schoolers has been classified as MVPA [22,54,55], it is likely that during a 30-minute session, the children will spend at least 10 minutes in MVPA. This will contribute towards the amount of MVPA required for health benefits.

There will be two phases to the intervention. The initiation phase (10 weeks) will be facilitated by parent involvement and/or early-years workers employed by schools/Children’s Centres. These facilitators will undertake a 2-hour training session using the PiP manual and will be accompanied during the first session by the researcher (SB) who developed the PiP intervention. Subsequent telephone support from the researcher will be offered to facilitators during the intervention period. The PiP session in the initiation phase consists of two, 5- minute structured parent-and-child play sessions and 20 minutes of free play. Further details of the intervention are given in Table 1. During the maintenance phase (20 weeks) playgrounds will remain available six times a week (three mornings and three afternoons) to parents and their pre-school children for 30 minutes at specific times coinciding with the so-called school run, allocated by the school/Children's Centre. For the purposes of the pilot trial, the facilitator (parent involvement and/or early-years worker) from the initiation phase will take a register of attendees at the beginning of the sessions but will not give formal supervision during the session. Table 1 illustrates the content, evidence for the content and associated behavioural change techniques for both the initiation and maintenance phase of the intervention.

**Table 1:** Content of the initiation and maintenance phases of the PiP intervention, the evidence to support the content and the behavioural change techniques used

**Content**	**Initiation (I) maintenance (M) phase**	**Evidence to support content**	**Behavioural change technique**
Provision of playground area at a time which coincides with families' daily routines (dropping off/picking up at school/nursery)	I, M	SCT (environmental factor) ^24, 25^	Environmental changes
		Focus group reports: activity sessions need to fit into other daily routines ^*47*^	
Provision of outdoor play equipment during sessions	I, M	SCT (environmental factor) ^24, 25^	Environmental changes
Facilitator to give telephone support and encourage families to attend the sessions	I, M	SCT (behavioural factor) ^24, 25^	Social processes of support/encouragement
Group sessions	I, M	Recommendation for pre-school obesity interventions ^23^	Social processes of support/encouragement
Supervision at the session from the facilitator	I	Focus group reports: parents need extra support to feel confident and safe playing with their children outside ^47^	Social processes of support/encouragement
Facilitator to encourage children to be physically active and give rewards (praise and “well done” stickers)	I	SCT (behavioural factors) ^24, 25,^ Recommendation for pre-school obesity interventions ^35^	Social processes of support/encouragement
Facilitators support parents to give positive reinforcement to their children’s physical activity	I	SCT (behavioural factors) ^24, 25^ Parent involvement is important for children’s behavioural change ^33, 34, 35^	Modelling Social processes of support/encouragement Increasing skills Rehearsal of skills
Facilitator to encourage outdoor play between parent and child outside of the intervention	I	CMO report ^13, 35^	Social processes of support/encouragement
Facilitator to provide information (verbally), give leaflets and facilitate parent discussions on the link between physical activity and health, guidelines for physical activity and sedentary behaviour in under 5-year-olds	I	SCT (personal factors) ^24, 25^	Provide information regarding behaviour
		Recommendation for pre-school obesity interventions ^23^	
			Information on consequences
			Social processes of support/encouragement
Ideas for active play provided and activity is playful	I (facilitator) M (instruction cards)	SCT (personal factors) ^24, 25^	Increasing skill
		CMO report ^13,^ Recommendation for pre-school obesity interventions ^35^	Skill rehearsal
			Modelling
			Graded tasks
Facilitator teaches two 5-minute structured parent and child games to develop child’s observational learning, locomotor, stability and object control skill	I	SCT (personal factors) ^24, 25^	Increasing skill
		CMO report ^13^	Skill rehearsal
			Modelling
			Graded tasks
Facilitator modifies play activities to suit the different needs of children in the group	I	CMO report ^13^ Recommendation for pre-school obesity interventions ^35^	Graded tasks
Children have 20 minutes free play	I	CMO report ^13^	Environmental changes
Facilitators to ensure regular infusions of different play equipment during free play	I	Recommendation for pre-school playground interventions ^33^	Environmental changes
Play equipment given out once a week to support families to play at home	I (3 schools only)	SCT (environmental factors and behavioural factors) ^24, 25^	Rewards/incentives
			Skill rehearsal
		CMO report ^13^	

PiP, pre-schoolers in the playground; SCT, social cognitive theory; CMO, Chief Medical Officer, superscript numbers denote the supporting reference.

### Outcome measures

In order to assess the feasibility of a full-scale cluster RCT of the PiP intervention, this pilot trial will examine recruitment rates, attendance and attrition, acceptability of the trial procedures and of the PiP intervention to parents, fidelity of intervention implementation; and capability and capacity for schools to deliver the intervention. Health outcomes and the feasibility of the tools used to measure these outcomes will also be assessed.

### Recruitment

The following will be recorded:

● Number of eligible children screened and agreed or declined further contact about the study

● Number of exclusions

● Number of parents of children who agreed to further contact about the study and are contacted or unable to be contacted

● From those parents who are contacted, the number who declined, missed the recruitment window (that is, the intervention/control period had already begun before they arranged an appointment for baseline measures) or were given an appointment for baseline measures

● Number that fail to attend the appointment for baseline measures, attend the appointment and do not consent, or attend and give consent

### Attendance and attrition

The following will be collected:

● Attendance rates at PiP sessions

● Who attends the PiP session with the child

● Attendance to measurement sessions (at baseline, 10, 30 and 52 weeks)

● Attrition (to both PiP and measurement sessions)

### Acceptability of the trial and the PiP intervention to parents

A purposive sample of 20 parents will be recruited to the qualitative component of the study. Selection will be on the basis of role (for example, mother, father, grandparent, or carer), ethnicity and whether an attender or drop-out. We will select 15 parents/carers from the intervention arm and five from the control arm. This maximum variation sampling approach [56] will ensure a wide range of views are collected. Parents will be interviewed after completing their 10 week data collection. Semi-structured interviews will explore the acceptability of PiP (intervention only) and their experience of taking part in the trial e.g. randomisation, measurement tools, and incentives (intervention and control). All interviews will be conducted using a topic guide to ensure consistency, although the format will be flexible in order to allow participants to generate naturalistic data on what they see as important. They will be audio-recorded digitally and transcribed verbatim.

### Fidelity of intervention implementation

Fidelity will be assessed in line with guidance from the NIH Behavior change Consortium [57]. To assess the fidelity of training for PiP facilitators, the trainer will be asked to reflect after sessions and complete a short form detailing the percentage of training delivered exactly as intended, and record any adaptations made to training. PiP facilitators will also complete a short evaluation form at the end of the training session to ensure provider skill acquisition.

To monitor the delivery of the intervention three sessions at each intervention school (two in the initiation and one in the maintenance phase) will be observed. A standardised form will be used for each observation where the observer will score PiP facilitators on a scale of 1 to 4 (1 being poor adherence to the intervention protocol and 4 being complete adherence to intervention protocol) for five key intervention factors (delivery of PiP as per the manual, supervision, support given to parents, encouragement of children, and infusion of play equipment). To monitor the number of sessions provided each week during the initiation phase of the intervention, PiP facilitators will be asked to complete a short form at the end of each session detailing whether the session was provided, the number of attendees to the session and the structured play activities provided at the session.

### Capability and capacity for delivery

At 10 weeks, the parent involvement/early-years workers who have delivered the intervention will be interviewed to explore the feasibility of the delivery and help refine the nature and content of intervention. At 30 weeks (after the maintenance phase) we will conduct telephone interviews with head teachers at schools in the intervention arm to explore their views on the acceptability of the intervention within the school setting, in particular the impact of PiP upon the school day. Both sets of interviews will be audio-recorded digitally and transcribed verbatim. A description of each school’s playground environment including size, availability of equipment/facilities/markings will be recorded along with the schools local environment (for example, proximity to other outdoor play space).

### Health outcomes

The following outcomes will be assessed at baseline, 10 weeks, 30 weeks and 52 weeks: for the child, daily time spent in physical activity, body mass index (BMI), waist and upper-arm circumferences and health-related quality of life; for the parent, health-related quality of life, self-efficacy and wellbeing.

#### Physical activity

Children will wear a triaxial, Actigraph GT3X+ accelerometer, (15 second epochs; Actigraph Pensacola, FL, USA) on a belt around their waist (anterior to the iliac crest) during waking hours for six days, including at least one weekend day [58]. Parents will be provided with a diary to record the times that the accelerometer is put on and taken off their child. Data will be downloaded and reduced using Actilife software version 5 (Actigraph). Non-wear time will be defined as consecutive zero counts of ≥ 10 minutes [59]. Parent-completed wear-time logs will be checked for periods when the monitor was not worn, and will be matched against Actigraph data. To determine the minimum wear time (that is, daily-wear hours and number of wear days) required to achieve a reliable estimate of habitual physical activity, that is, intraclass correlation coefficient (ICC) value ≥ 0.8 [60], the Spearman-Brown prophecy formula will be applied to participants' baseline accelerometer data.

Vertical axis activity counts will be converted to time spent sedentary (≤ 37 counts per 15-second epoch [61]) in light physical activity, (38 to 419 counts per 15-second epoch in MVPA (≥ 420 counts per 15-second epoch and in total physical activity (light to vigorous; ≥ 38 counts per 15-second epoch). The cutpoints derived by Pate et al. [61] for children aged 3 to 5 years old were selected as they have been cross-validated under free-living conditions and have been found to have better agreement with directly observed physical activity than other published Actigraph intensity cutpoints in children aged 16 to 35 months [62]. Daily physical activity energy expenditure will be calculated using the Actilife software (Version 6). Additionally, the percentage of children meeting physical activity guidelines of 180 minutes per day [28] (including light, moderate and vigorous physical activity) will be calculated.

### Anthropometry

Body mass will be measured in standard clothing conditions (lightweight clothing), using regularly calibrated Seca electronic scales (Medical Scales and Measuring Systems, Birmingham UK). Height will be measured using a Leicester Height Measure (Harlow Healthcare Limited,HarlowUK). Waist circumference will be measured at the midpoint between the lowest rib and the iliac crest. Upper arm circumference will be measured at the midpoint between the acromion process of the scapula and the olecranon process. Body mass and height will be used to calculate BMI, these will be converted to age- and sex-adjusted *z*-scores relative to the World Health Organization (WHO) 2006 growth standard using the least mean squares (LMS) method. Percentage of overweight children in each arm (intervention or control) will also be recorded and defined as having a BMI z-score greater than +1.04 (= 85th centile).

### Health related quality of life and well-being

The child’s health-related quality of life will be assessed using the Paediatric Quality of Life questionnaire (PedsQL; infant or 2- to 4-year-old version). The PedsQL infant and toddler scales are reliable, valid and have good internal consistency (≥ 0.7) [63]. Parental health-related quality of life will be assessed using the EQ5D, this self-complete standardised instrument is validated for use and is reliable for adult populations (convergent validity: Spearman rank order coefficients with WHO-5 sum-scores = 0.49; [64]). Parental wellbeing will be assessed using the Comprehensive Quality of Life Scale-Adult (ComQol-A5) [65]; it is a valid and reliable scale (Cronbach’s Alpha = 0.39 for all seven domains [66]) that is designed to be used with any section of the adult population. Parental self-efficacy will be assessed using the General Self Efficacy Scale; the scale is valid for use in a general adult population and has excellent reliability (Cronbach’s Alpha > 0.80) [67].

### Injuries and health service use

Number and type of injuries sustained during the intervention/usual care period and the consequence of the injury (that is, medical attention from General Practitioner, or hospital visit) and service use (health and social care) will be reported by parents and recorded at each assessment point using previously developed questionnaires.

### Feasibility of the measurement tools

The feasibility of using all of the measurement tools in a full trial will be assessed; this is important to predict the success of a full-scale trial and to ensure that the trial is not too burdensome for participants. Using accelerometry to measure physical activity will be assessed by examining the number of participants who meet the criteria for inclusion in data analysis. The feasibility of measuring BMI, and waist and upper arm circumference will be assessed by the percentage of data collected. The feasibility of using the following questionnaires will be assessed by examining completion rates: for children, infants (13 months to 2 years old) and toddlers (2 to 4 years old), the PedsQL; for parents, the EQ5D, ComQol-A5 [67] and General Self Efficacy Scale [67].

### Data to inform sample size calculation

An important aspect of the pilot study is to collect data to estimate the sample size required for a full RCT. The pilot RCT will provide data on the variability of outcome measures; estimates of effect sizes between control and intervention, and estimates of ICCs which are currently unavailable from other studies. It will also provide a more accurate estimate of the follow-up rate.

### Data analysis

#### Statistical analysis

For both arms the numbers of schools and children screened, randomly assigned, receiving PiP or usual care, completing the study protocol, and providing outcome data will be summarised. The number of children withdrawing from the intervention and/or the trial, and where available the reasons for withdrawal, will be summarised by arm. For each data collection point the number of non-attenders will be calculated for each arm and attendance rates compared.

Whilst the main aim of this study is to establish practicality, feasibility, recruitment rates and samples size in order to inform a full-scale trial, and although it is unlikely that the small sample size will result in effectiveness being established, the primary outcome for a full trial, daily MVPA, will be analysed to mimic practice in a full-scale trial. Results from this analysis will be treated as preliminary and interpreted with caution [68,69]. As the number of clusters is low, cluster summary statistics will be utilised rather than multi-level modelling [70,71]. The analysis will be carried out using the school as the unit of analysis and the mean MVPA per day for the individuals in the school as the outcome variable. A weighted linear regression model will be used to compare the intervention and usual-care arms weighted by the number of participants followed up in each cluster and adjusted for the baseline average MVPA per day for each cluster. We acknowledge that participation in a physical activity trial may in effect be an intervention in itself for the control group. Therefore the difference in health outcomes (physical activity and anthropometry) between the intervention and control arms will provide meaningful data on the potential effectiveness of the intervention. We will assess whether playground environment and local environment have a significant impact upon intervention uptake, attendance and changes in levels of physical activity. If they are found to be significant we will consider stratifying schools according to their environment in the full trial.

#### Health economics analysis

The possibility of using routine databases to capture relevant resource use will be explored and this will be supported by parent and child quality-of-life questionnaires (PedsQL, EQ5D and ComQol-A5), and parent-reported injuries and service use at each assessment time point.

If the data can be collected reliably, an exploratory evaluation will assess whether the addition of the PiP intervention is likely to be cost-effective in the pilot trial. It is acknowledged that the benefits of PiP may be generated over a longer time frame than captured by the trial. We will thus conduct a within-trial analysis and, if possible, an analysis of the costs and benefits associated with the intervention modelled over a more appropriate time horizon. A key part of the economic analysis will be to identify potential sources of data to populate a longer term economic model. In addition, the delivery costs and the resource use associated with the intervention (for example, health service usage including General Practitioner visits, or Accident and Emergency attendances) will be estimated.

Unit costs estimated from the published literature and government sources will be presented. These unit costs will then be applied to the relevant resource use. Each total cost item will then be summed to generate a total cost per trial participant. For this exploratory analysis, where it is considered appropriate, cost and quality-adjusted life year (QALY) data will be synthesised to generate an incremental cost-effectiveness ratio (ICER) where the additional cost of the intervention is formally compared with the additional benefit. Probabilistic sensitivity analyses will be conducted to characterise the uncertainty around the adoption decision (depicted using cost-effectiveness acceptability curves) and to assess the potential and value of further research in this area.

The trial assesses a large number of primary and secondary outcomes. Hence, for a complete picture of the trials results, all the costs and outcomes will be presented in a descriptive and disaggregated way. A review of the literature will be conducted to establish whether it is possible to make links between short-term outcomes measured in the trial and longer-term quality of life, and therefore populate the model.

#### Qualitative analysis

The qualitative interview data (from parents, PiP facilitators and head teachers) will be analysed using the constant comparison method through thematic coding of the data [72]. Coding will take place using a combination of a prior themes and emergent themes. Negative cases will actively be sought throughout the analysis and emerging ideas and themes modified in response [73]. ATLAS-ti software will aid data handling.

## Discussion

This pilot cluster RCT will assess the feasibility of a parent and child playground-based physical activity intervention for pre-school children in order to inform whether or not a fully powered trial should be undertaken. It is anticipated that results of this pilot trial will be published in spring 2015.

### Trial status

Recruitment for Wave 1 and 2 of the PiP pilot cluster RCT is complete; recruitment for Wave 3 is underway (April 2013).

## Abbreviations

BMI: Body mass index; CLAHRC: Collaborations for leadership in applied health research and care; CMO: Chief medical officer; ComQol-A5: Comprehensive quality of life scale-adult; EQ5D: Euroquol 5D; ICC: Intraclass correlation coefficient; ICER: Incremental cost effectiveness ratio; IMD: Index of multiple deprivation; LMS: Least mean squares; MVPA: Moderate-to-vigorous physical activity; NIHR: National institute for health research; PedsQL: Paediatric quality of life; PiP: Pre-schoolers in the playground; QALY: Quality-adjusted life year; RCT: Randomised controlled trial; SCT: Social cognitive theory; WHO: World Health Organization.

## Competing interests

The authors declare that they have no competing interests.

## Authors’ contributions

All authors have made substantial contributions to the concept and design of the study. In particular: CJ, HA, SA and JW contributed expertise on trial design; SBa, DB, SA, HM, CS and SBr designed and developed the PiP intervention; SB, AR, GR, KP, CO’M and HM selected and justified the outcome measure tools; CH contributed statistical analysis expertise and GR the health economic evaluation; SBa drafted the main body of the manuscript; CS and KP contributed to the background, CJ the trial design, CH the statistical analysis, and GR the health economic section. All authors provided revisions on the content of the paper and have given final approval for publication. All authors read and approved the final manuscript.
